# Leukocyte telomeres are longer in African Americans than in whites: the National Heart, Lung, and Blood Institute Family Heart Study and the Bogalusa Heart Study

**DOI:** 10.1111/j.1474-9726.2008.00397.x

**Published:** 2008-08

**Authors:** Steven C Hunt, Wei Chen, Jeffrey P Gardner, Masayuki Kimura, Sathanur R Srinivasan, John H Eckfeldt, Gerald S Berenson, Abraham Aviv

**Affiliations:** 1 Cardiovascular Genetics Division, University of Utah School of MedicineSalt Lake City, UT, USA (NHLBI Family Heart Study); 2 Tulane Center for Cardiovascular Health, Tulane University Health Sciences CenterNew Orleans, LA, USA (The Bogalusa Heart Study); 3 Center of Human Development and Aging, University of Medicine and Dentistry of New Jersey, New Jersey Medical SchoolNewark, NJ, USA; 4 Department of Laboratory Medicine and Pathology, University of MinnesotaMinneapolis, MN, USA (NHLBI Family Heart Study)

**Keywords:** age, demography, gender, leukocyte, race, telomere

## Abstract

Leukocyte telomere length (LTL) is ostensibly a bio-indicator of human aging. Here we report that African Americans have longer LTL than whites. We studied cross-sectionally 2453 individuals from the National Heart, Lung, and Blood Institute (NHLBI) Family Heart Study (age = 30–93 years) and the Bogalusa Heart Study (age = 19–37 years), comprising 1742 whites and 711 African Americans. We measured LTL by Southern blots of the terminal restriction fragments length. In 234 participants, telomere repeats were also measured by quantitative polymerase chain reaction (qPCR). Adjusted for age and body mass index (BMI), the respective leukocyte telomere lengths (mean ± SEM) were considerably longer in African Americans than in whites both in the Family Heart Study (7.004 ± 0.033 kb vs. 6.735 ± 0.024 kb, *p* < 0.0001) and the Bogalusa Heart Study (7.923 ± 0.063 kb vs. 7.296 ± 0.039 kb, *p* < 0.0001). We confirmed the racial effect on LTL by qPCR (3.038 ± 0.565 T/S units for African Americans vs. 2.714 ± 0.487 T/S units for whites, *p* < 0.001). Cross-sectionally, sex- and BMI-adjusted LTL became shorter with age (range 19–93 years) at a steeper slope in African Americans than in whites (0.029 kb year^−1^ vs. 0.020 kb year^−1^, respectively, *p* = 0.0001). We suggest that racial difference in LTL arises from a host of interacting biological factors, including replication rates of hematopoietic stem cells.

## Introduction

Mounting evidence suggests that leukocyte telomere length (LTL) is a bio-indicator of human aging, cardiovascular aging in particular. LTL is heritable (Slagboom *et al*., 1994; Jeanclos *et al*., 2000; Vasa-Nicotera *et al*., 2005; Andrew *et al*., 2006), although it is unknown how much of this heritability relates to birth LTL and the rate of its shortening from birth onward. Age-dependent LTL shortening is due to successive divisions of hematopoietic stem cells (HSCs) and progenitor cells (PCs) that form peripheral leukocytes. Inflammation and oxidative stress – two central elements in the biology of aging and aging-related diseases (Finch & Crimmins, 2004; Balaban *et al*., 2005) – were reported to be associated with LTL (Aviv *et al*., 2006a; Demissie *et al*., 2006; Bekaert *et al*., 2007; Fitzpatrick *et al*., 2007). Inflammation entails an increase in number and diminished biological life of leukocyte subsets in the circulation, which would heighten the demand on HSCs/PCs to replicate, a phenomenon expressed in an accelerated telomere attrition and ultimately shortened LTL. Oxidative stress heightens the loss of telomere repeats per cell division (Sitte *et al*., 1998; Saretzki *et al*., 1999; Tchirkov & Lansdorp, 2003). The compounded effect of inflammation/oxidative stress on the paces of both aging and LTL attrition conceivably explains the shortened LTL observed in individuals with aging-related diseases, particularly atherosclerotic cardiovascular (CV) disease (Brouilette *et al*., 2003; Cawthon *et al*., 2003; Benetos *et al*., 2004; Martin-Ruiz *et al*., 2005; Matsubara *et al*., 2006; Brouilette *et al*., 2007; Fitzpatrick *et al*., 2007; van der Harst *et al*., 2007), which is strongly linked to inflammation (Hansson & Libby, 2006).

The paradigm that links LTL to CV disease draws on data derived mainly from non-African American populations. But the developmental pattern of CV disease differs between African Americans and whites. For instance, African Americans are more prone to heart failure due to hypertension (Yancy, 2005; Whittle *et al*., 2006), but they have considerably less coronary artery calcification than do whites (Tang *et al*., 1995; Yan *et al*., 2006; McClelland *et al*., 2006; Aiyer *et al*., 2007; Loria *et al*., 2007). We therefore explored in two ongoing studies, the National Heart, Lung, and Blood Institute (NHLBI) Family Heart Study (FHS) and the Bogalusa Heart Study (BHS), the racial effect on LTL.

## Results

**Fig. 1:**
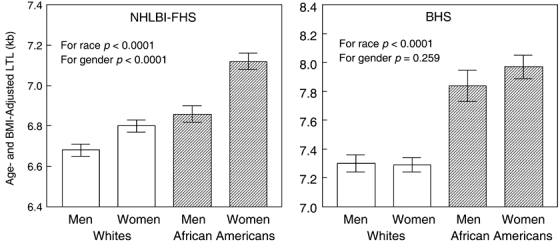
Age- and body mass index-adjusted leukocyte telomere length (LTL) in the NHLBI Family Heart Study (FHS) and the Bogalusa Heart Study (BHS), based on terminal restriction fragment lengths, determined in the entire sample by *Hin*fI/*Rsa*I restriction enzymes. The lower LTL values in the FHS than the BHS cohorts relate to the older age of the participants of the FHS.

Mean ages and BMI of whites and African Americans, by sex, are displayed in Table 1. In both the FHS and the BHS cohorts, LTL, measured by Southern blots of the terminal restriction fragment (TRF) products of *Hin*fI/*Rsa*I, was significantly longer in African Americans than in whites (Table 2; Fig. 1). As LTL was inversely correlated to BMI (*r* = –0.071, *p* = 0.002), we adjusted LTL for the BMI. For the FHS cohort, sex-specific differences in age- and BMI-adjusted LTL between African Americans and whites were 180 base pairs (bp) for men and 320 bp for women; for the BHS cohort, these differences amounted to 500 bp for men and 680 bp for women. Heritability of LTL in whites was 0.69 ± 0.03, while in African Americans it was 0.78 ± 0.09, both significant at *p* < 0.0001. There was no statistically significant difference in heritability between African Americans and whites.

**Table 2:** Leukocyte telomere parameters by race and sex in the two cohorts, in which leukocyte telomere length (LTL) was measured using restriction enzymes *Hin*fI/*Rsa*I

	Whites		African Americans			
	Men	Women	Men	Women	*p* gender	*p* race
FHS						
LTL	6.67 ± 0.03	6.77 ± 0.03	6.93 ± 0.05	7.16 ± 0.04	< 0.0001	< 0.0001
Age-adjusted LTL	6.68 ± 0.03	6.81 ± 0.03	6.86 ± 0.05	7.10 ± 0.03	< 0.0001	< 0.0001
Age- and BMI-adjusted LTL	6.68 ± 0.03	6.80 ± 0.03	6.86 ± 0.04	7.12 ± 0.04	< 0.0001	< 0.0001
BHS						
LTL	7.28 ± 0.06	7.32 ± 0.05	7.81 ± 0.10	7.95 ± 0.08	0.5087	< 0.0001
Age-adjusted LTL	7.29 ± 0.06	7.31 ± 0.05	7.83 ± 0.11	7.93 ± 0.08	0.5241	< 0.0001
Age- and BMI-adjusted LTL	7.30 ± 0.06	7.29 ± 0.05	7.84 ± 0.11	7.97 ± 0.08	0.2590	< 0.0001

BMI, body mass index; BHS, Bogalusa Heart Study; FHS, Family Heart Study.

**Table 1:** Means (± standard deviation) of age and body mass index of the two cohorts

	Whites		African Americans		Entire sample	
	Men	Women	Men	Women	Men	Women
FHS	(*n* = 610)	(*n* = 785)	(*n* = 195)	(*n* = 378)	(*n* = 805)	(*n* = 1163)
Age (years)	57.3 ± 13.6	58.5 ± 13.1	52.4 ± 10.6	54.0 ± 10.9	56.1 ± 13.1	57.1 ± 12.6
Body mass index (kg m^−2^)	29.4 ± 4.7	28.4 ± 5.9	30.6 ± 6.1	34.1 ± 7.8	29.7 ± 5.1	30.2 ± 7.1
BHS	(*n* = 140)	(*n* = 207)	(*n* = 44)	(*n* = 94)	(*n* = 184)	(*n* = 301)
Age (years)	30.9 ± 4.6	30.1 ± 4.8	31.6 ± 3.9	29.4 ± 5.0	31.1 ± 4.4	30.0 ± 4.9
Body mass index (kg m^−2^)	27.8 ± 5.3	25.5 ± 6.1	28.6 ± 8.2	30.6 ± 8.6	28.0 ± 6.1	27.1 ± 7.4

BHS, Bogalusa Heart Study; FHS, Family Heart Study.

In the FHS cohort, women of both races displayed significantly longer LTL than men (Table 2; Fig. 1). The sex-related differences in age- and BMI-adjusted LTL for this cohort were 120 bp for whites and 260 bp for African Americans. However, no such difference was noted between women and men of both races in the relatively younger BHS cohort (Table 2; Fig. 1). Further details regarding LTL results derived from the *Hin*fI/*Rsa*I digest are summarized in Table 2.

**Fig. 2:**
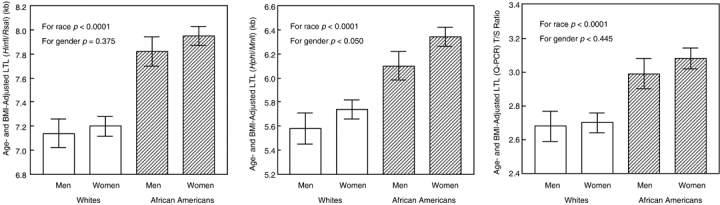
Age- and body mass index-adjusted leukocyte telomere length (LTL) in a subset of the Bogalusa Heart Study (BHS) based on terminal restriction fragment lengths, determined by using restriction enzymes *Hin*fI/*Rsa*I and *Hph*I/*MnI*I, and by quantitative polymerase chain reaction. Figure displays results from 72 men and 162 women, equally divided by race.

In most clinical studies, the restriction enzymes for TRF analysis typically comprise *Hin*fI/*Rsa*I. In principle, longer LTL in African Americans than in whites may arise from race-related polymorphisms in length or the nearest restriction site proximal to the canonic (TTAGGG) stretch of the telomeric repeats. For this reason we also measured in a subset of the BHS cohort TRF length using the restriction enzymes *Hph*I/*MnI*I. Both restriction enzymes *Hin*fI/*Rsa*I and *Hph*I/*MnI*I yield an admixture of TRFs comprising primarily canonical but also some noncanonical telomere repeats at the proximal region of the telomeres. However, *Hph*I/*MnI*I cut the DNA at TGAGGG and TCAGGG, yielding shorter TRFs than those generated by *Hin*fI/*Rsa*I (Allshire *et al*., 1989; Baird *et al*., 2006). This is shown in Fig. 2 and Table 3 (and in Supplementary Fig. S1). In this subset, we also measured telomeric DNA content by quantitative polymerase chain reaction (qPCR) analysis, which strictly quantifies telomere repeats. Regardless of the method used, African Americans displayed longer LTL (or a higher T/S ratio) than whites (Fig. 2; also see Table 3). We note that the qPCR method (Cawthon, 2002) has a higher CV% than the TRF length analysis, which might limit detection of small variation in telomere length (Aviv *et al*., 2006b). However, given the considerable difference in LTL between African Americans and whites, the qPCR measurements confirmed the findings of the TRF length analysis. Interestingly, analysis of the TRF products generated by the *Hph*I/*MnI*I digest showed a significant sex effect on LTL in the BHS.

**Table 3:** Leukocyte telomere parameters in a subset of the Bogalusa Heart Study by race and sex in which leukocyte telomere length (LTL) and telomere repeats were measured using restriction enzymes *Hin*fI/*Rsa*I and *Hph*I/*MnI*I, and by quantitative polymerase chain reaction (qPCR)

	Bogalusa Heart Study					
	Whites		African Americans			
	Men	Women	Men	Women	*p*-value gender	*p*-value race
LTL (*Hin*fI/*Rsa*I) (kb)						
Unadjusted	7.15 ± 0.13	7.25 ± 0.08	7.80 ± 0.11	7.91 ± 0.08	0.4702	< 0.0001
Age-adjusted	7.16 ± 0.12	7.24 ± 0.08	7.81 ± 0.12	7.91 ± 0.08	0.3878	< 0.0001
Age- and BMI-adjusted	7.14 ± 0.12	7.20 ± 0.08	7.82 ± 0.12	7.95 ± 0.08	0.3748	< 0.0001
LTL (*Hph*I*/MnI*I) (kb)						
Unadjusted	5.59 ± 0.13	5.79 ± 0.08	6.07 ± 0.13	6.32 ± 0.08	0.0292	< 0.0001
Age-adjusted	5.61 ± 0.12	5.78 ± 0.08	6.08 ± 0.12	6.31 ± 0.08	0.0637	< 0.0001
Age- and BMI-adjusted	5.58 ± 0.13	5.74 ± 0.08	6.10 ± 0.12	6.34 ± 0.08	0.0503	< 0.0001
qPCR LTL (T/S units)						
Unadjusted	2.71 ± 0.08	2.71 ± 0.05	3.01 ± 0.10	3.05 ± 0.06	0.7923	< 0.0001
Age-adjusted	2.69 ± 0.09	2.72 ± 0.06	2.99 ± 0.08	3.06 ± 0.06	0.4788	< 0.0001
Age- and BMI-adjusted	2.68 ± 0.09	2.70 ± 0.06	2.99 ± 0.09	3.08 ± 0.06	0.4449	< 0.0001

BMI, body mass index.

We also obtained the overall and differential counts of leukocytes and their subsets, including neutrophils, in the majority of participants of the BHS whose telomere parameters were measured (Supplementary Table S1). Although African Americans showed significantly lower leukocyte and neutrophil counts than did whites, no relationships were observed between these counts and LTL in African Americans and whites, independently or jointly.

**Fig. 3:**
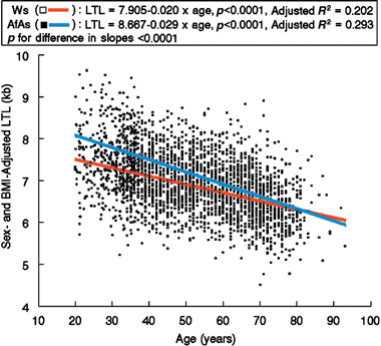
Sex- and body mass index-adjusted leukocyte telomere length (LTL) vs. age for African Americans and whites from the NHLBI Family Heart Study (FHS) and the Bogalusa Heart Study (BHS) combined.

Figure 3 displays sex- and BMI-adjusted LTL vs. age from the combined data set of the FHS and BHS, using LTL derived from *Hin*fI/*Rsa*I digest. African Americans had higher LTL at nearly all ages. However, sex- and BMI-adjusted LTL became shorter with age at a steeper slope in African Americans (0.029 kb year^−1^) than in whites (0.020 kb year^−1^) (*p* = 0.0001). The slope of LTL vs. age did not differ by sex.

## Discussion

The central finding of this study is that African Americans have considerably longer LTL than whites, at least up to age 80. In this study, we observed a sex effect on LTL in the NHLBI FHS, but in the BHS, the sex effect was found only in the products of the *Hph*I/*MnI*I. A potential explanation for the difficulty in detecting the sex effect may be the much younger age of the BHS participants. In the FHS and in previous studies that found longer LTL in women than men (Jeanclos *et al*., 2000; Benetos *et al*., 2001; Nawrot *et al*., 2004; Vasa-Nicotera *et al*., 2005; Bekaert *et al*., 2007), the subjects were much older than those in the BHS.

LTL is equal in African American and white newborns (Okuda *et al*., 2002). As African Americans in their 20s already display longer LTL than their white peers, the racial gap in LTL might relate to factors that define leukocyte telomere dynamics during the formative years. These include the proliferative rates of HSCs/PCs. African Americans and other individuals of African ancestry display lower leukocyte and neutrophil counts than do whites (Haddy *et al*., 1999; Bain *et al*., 2000; Phillips *et al*., 2000; Mayr *et al*., 2007), a finding we confirmed in the BHS. The neutrophils in peripheral blood are distributed into two pools; namely, circulating cells and marginated cells that adhere to the endothelium in postcapillary venules (Athens *et al*., 1961). Physiological neutropenia in individuals of African ancestry is not due to increased margination of neutrophils (Athens *et al*., 1961; Bain *et al*., 2000; Phillips *et al*., 2000). It is therefore likely to arise from fewer replications of HSCs/PCs. The longer LTL in African Americans than in whites is consistent with this premise.

Telomere length is shorter in neutrophils than in T lymphocytes in young individuals and vice versa in older individuals (Weng, 2001). For the following reasons, we suggest that in and of itself this phenomenon does not explain the racial difference in LTL: First, LTL was found to be longer in African Americans than in whites at a wide age range that encompasses most of adult life. Second, there are considerable interindividual variations in LTL at birth (Okuda *et al*., 2002; Akkad *et al*., 2006) and thereafter (Jeanclos *et al*., 2000; Benetos *et al*., 2001; Gardner *et al*., 2005; Nawrot *et al*., 2005; Valdes *et al*., 2005; Vasa-Nicotera *et al*., 2005; Bekaert *et al*., 2007; Njajou *et al*., 2007). These interindividual variations in telomere length far exceed the variation in telomere length among cell types within the individual, because telomere length is synchronized (equivalent) in different tissues and cells in the fetus (Youngren *et al*., 1998) and the newborn (Okuda *et al*., 2002), and partially synchronized at any age (Butler *et al*., 1998; Martens *et al*., 1998; von Zglinicki *et al*., 2000; Takubo *et al*., 2002; Gardner *et al*., 2007). It follows that individuals with relatively long (or short) telomere length in one cell type have long (or short) telomere length in other cell types. Regarding telomere dynamics in leukocytes, LTL reflects birth telomere length and replicative history of HSCs/PCs. At any given time, and for whatever reason and duration, an altered demand by a leukocyte subset on PCs to increase or diminish their divisions would ultimately impact telomere dynamics in HSCs and therefore telomere length in all leukocytes. It is unlikely therefore that the racial differences in LTL may be simply explained by a subset of leukocytes. Third, in the BHS we found no association of LTL with the numbers of either leukocytes or neutrophils (Supplementary Table S1). This is not unexpected, as LTL is a record of the replicative history of HSCs/PCs over the individual's lifetime until sample collection, while the leukocyte and neutrophil counts reflect a ‘snapshot’ of the peripheral leukocytes at the time of sample collection.

Although diminished HSC/PC replication might explain the longer LTL in African Americans than in whites during early adulthood, additional factors apparently tip the balance towards narrowing the racial gap in LTL later in life. Theoretical considerations suggest that telomere attrition rate in cultured cells is proportional to telomere length (op den Buijs *et al*., 2004), perhaps because longer telomeres are a greater target to free radicals. All other things being equal, the relatively longer LTL in adult African Americans may account in part for a higher rate of LTL shortening. In addition, African Americans exhibit increased prevalence of risk factors – not only for CV disease (whose pattern may not be the same as in whites), but also for other potentially deleterious conditions, e.g. low social status and unhealthy lifestyle (Otten *et al*., 1990), which were shown to be associated with shortened LTL (Cherkas *et al*., 2006; Bekaert *et al*., 2007). Despite the racial differences in LTL and the rate of its shortening during adulthood, we found LTL to be heritable in both races.

Might the longer LTL in African Americans than whites provide clues with regard to racial differences in life expectancy in the USA? Until recently, a controversy existed about the relation between LTL and mortality/survival in elderly persons (Cawthon *et al*., 2003; Martin-Ruiz *et al*., 2005; Bischoff et al., 2006; Harris *et al*., 2006; Honig *et al*., 2006). Of these, two studies (Cawthon *et al*., 2003; Honig *et al*., 2006) showed an association between mortality and LTL. The other three did not. We recently addressed this question in the Longitudinal Study of Aging Danish Twins, which comprises same-sex, elderly twins, whose mortality/survival was monitored for 10 years after leukocyte collection. The same-sex twin model is the most optimal to study the connection between mortality and LTL because it controls for genes, rearing environment, sex and age, all of which might impact LTL. Using Southern blot analysis approach, it was found that the co-twin with the shorter telomere parameters was likely to die first by the 4th year of the 10 years follow-up (Kimura *et al*., in press). Another study reported similar findings in same-sex Swedish twins (Bakaysa *et al*., 2007).

The mechanisms that define the upper boundary of lifespan – namely, the survival of elderly individuals who withstood or did not suffer aging-related diseases during midlife – might not be the same as those that determine aging in the general population (Kimura *et al*., 2007). However, assuming that LTL is associated with lifespan in the elderly, there are curious observations regarding differences in mortality/survival between African Americans and whites. African Americans exhibit increased clustering of risk factors not only for CV disease but also other potentially lethal maladies; in the USA they display higher mortality rates and shorter life expectancy than whites (Otten *et al*., 1990). However, above the age of 70–80 years African Americans have been reported to exhibit lower mortality than whites (Markides & Machalek, 1984; Wing *et al*., 1985; Ford *et al*., 1990; Elo & Preston, 1994). This crossover phenomenon was challenged due to concerns that vital statistics data reflect systematic misreporting of age among elderly African Americans (Coale & Kisker, 1986; Preston *et al*., 1996). However, more recent work further supports the mortality crossover, at least with respect to coronary heart disease mortality (Corti *et al*., 1999). The etiology of this finding is unknown. The faster LTL shortening in African Americans would narrow the racial difference in LTL, but the vestigial effect of the LTL ‘advantage’ in African Americans throughout most of adult life might narrow the racial gap in mortality in the elderly.

A number of limitations of this study are noteworthy. First, this cross-sectional study is not as powerful as the longitudinal approach to examine leukocyte telomere dynamics. Second, our conclusions that LTL attrition rate is slower in African Americans than in whites between birth and early adulthood is based on indirect evidence. Third, our study focuses on the racial difference in LTL without factoring a host of race-associated circumstances that might account for the narrowing of the racial gap in LTL. However, the racial difference in LTL is of such a magnitude that it would probably eclipse the effect of any single environmental factor.

We propose that race and ethnicity should be assessed in future studies that explore the associations between diseases of aging, CV disease, in particular, and leukocyte telomere parameters.

## Experimental procedures

### The cohorts

The NHLBI FHS is a multicenter investigation of the genetic and epidemiologic basis of CV disease (Higgins *et al*., 1996). Between January 2002 and January 2004, 3359 family members were studied (2737 whites, 622 African Americans). The LTL data are derived from 1968 individuals from this cohort (1163 women, 805 men, 1395 whites and 573 African Americans) with an age range of 30–93 years.

The BHS is a long-term community-based epidemiologic study of early natural history of CV disease beginning in childhood from the semirural, biracial community of Bogalusa, LA (Bogalusa Heart Study, 1995). Between September 1995 and December 1996, 1420 individuals (1011 whites, 409 African Americans) were examined for CV risk factors. The LTL data were derived from 485 individuals of this cohort who had stored blood available (301 women, 184 men, 247 whites and 138 African Americans) with an age range of 19–37 years.

The study protocol was approved by Institutional Review Boards of centers that oversee each of the participating cohorts and each participant gave written informed consent.

### Telomere length measurements

The mean length of the TRFs, determined by Southern blot analysis, was used to measure LTL on DNA extracted from peripheral leukocytes. We obtained the mean TRF length in two ways: our standard method (Okuda *et al*., 2002) and an ‘overlay’ method (Vasan *et al*., in press), both utilizing *Hin*fI/*Rsa*I restriction enzymes. (Description of the ‘overlay’ method, used for DNA samples derived from the FHS, is provided in the Supplementary Appendix S1.) For the standard method, the coefficient of variation for duplicate samples, which were resolved on different gels, was 1.43% while for the overlay method it was 2.40%. The correlation between the two methods was *r* = 0.99, *p* < 0.0001 (*n* = 24). In a subset of participants, TRF length was also measured in DNA digested with restriction enzymes *Hph*I/*MnI*I. The TRF length derived from these restriction enzymes highly correlated with that derived from *Hin*fI/*Rsa*I (*r* = 0.814, *p* = 0.0001; Supplementary Fig. S1). In the same subset we also measured telomere repeats by qPCR, using minor modification (Gardner *et al*., 2007) of the method originally described by Cawthon (2002). The coefficient of variation for duplicate samples undergoing qPCR on different days was 6.10%. The laboratory that conducted the TRF length and qPCR assays was blinded to the identity of the subjects.

### Statistical analyses

The primary analysis consisted of comparing LTL parameters between African Americans and whites, and between men and women after adjustment for age and BMI. To investigate cross-sectional, race-specific associations of age with LTL, sex- and BMI-adjusted LTLs from each study were combined and analyzed by generalized estimating equations and an exchangeable correlation matrix (PROC GENMOD in SAS) to correct the estimated standard errors for the relatedness of the subjects within families. This correction prevents inflated significance levels of the tests used in the analyses presented. An age-by-race interaction term was included in the model to test if there were different age-related cross-sectional rates of LTL shortening between the two races. When significant, this interaction term was replaced by nesting age within race to estimate the two different slopes and their standard errors. Sex-by-age interactions with LTL shortening were not significant. Race-specific heritabilities of LTL in the FHS were obtained from SOLAR (Almasy & Blangero, 1998), adjusting for sex, age and specific gel on which the sample was run. Unless otherwise indicated, data are presented as mean ± SEM.

## Supplementary material

The following supplementary material is available for this article:

**Appendix S1** Terminal restriction fragment length analysis by the overlay method (Vasan *et al*. in press).

**Fig. S1** Relationship between leukocyte telomere length, obtained using *Hph*I/*MnI*I vs. leukocyte telomere length obtained using *Hin*fI/*Rsa*I.

**Table S1** Leukocyte differential counts in a subset of the Bogalusa Heart Study by race and sex

This material is available as part of the online article from: http://www.blackwell-synergy.com/doi/abs/10.1111/j.1474-9726.2008.00397.x (This link will take you to the article abstract).

## References

[b1] Aiyer AN, Kip KE, Marroquin OC, Mulukutla SR, Edmundowicz D, Reis SE (2007). Racial differences in coronary artery calcification are not attributed to differences in lipoprotein particle sizes: the heart strategies concentrating on risk evaluation (Heart SCORE) Study. Am. Heart J.

[b2] Akkad A, Hastings R, Konje JC, Bell SC, Thurston H, Williams B (2006). Telomere length in small-for-gestational-age babies. BJOG.

[b3] Allshire RC, Dempster M, Hastie ND (1989). Human telomeres contain at least three types of G-rich repeat distributed non-randomly. Nucleic Acids Res.

[b4] Almasy L, Blangero J (1998). Multipoint quantitative-trait linkage analysis in general pedigrees. Am. J. Hum. Genet.

[b5] Andrew T, Aviv A, Falchi M, Gardner JP, Lu X, Kimura M, Kato BS, Valdes AM, Spector TD (2006). Mapping genetic loci that determine leukocyte telomere length in a large sample of unselected, female sibling-pairs. Am. J. Hum. Genet.

[b6] Athens JW, Raab SO, Haaab OP, Mauer AM, Ashenbrucker H, Cartwright GE, Wintrobe MM (1961). Leukokinetic studies. III. The distribution of granulocytes in the blood of normal subjects. J. Clin. Invest.

[b7] Aviv A, Valdes A, Gardner JP, Swaminathan R, Kimura M, Spector TD (2006a). Menopause modifies the association of leukocyte telomere length with insulin resistance and inflammation. J. Clin. Endocrinol. Metab.

[b8] Aviv A, Valdes AM, Spector TD (2006b). Human telomere biology: Pitfalls of moving from the laboratory to epidemiology. Int. J. Epidemiol.

[b9] Bain BJ, Phillips D, Thomson K, Richardson D, Gabriel I (2000). Investigation of the effect of marathon running on leucocyte counts of subjects of different ethnic origins: relevance to the aetiology of ethnic neutropenia. Br. J. Haemotol.

[b10] Baird DM, Britt-Compton B, Rowson J, Amso NN, Gregory L, Kipling D (2006). Telomere instability in the male germline. Hum. Mol. Genet.

[b11] Bakaysa SL, Mucci LA, Slagboom PE, Boomsma DI, McClearn GE, Johansson B, Pedersen NL (2007). Telomere length predicts survival independent of genetic influences. Aging Cell.

[b12] Balaban RS, Nemoto S, Finkel T (2005). Mitochondria, oxidants, and aging. Cell.

[b13] Bekaert S, De Meyer T, Rietzschel ER, De Buyzere ML, De Bacquer D, Langlois M, Segers P, Cooman L, Van Damme P, Cassiman P, Van Criekinge WV, Verdonck P, De Backer GG, Gillebert TC, Van Oostveldt P, on behalf of the Asklepios investigators (2007). Telomere length and cardiovascular risk factors in middle-aged population free of overt cardiovascular disease. Aging Cell.

[b14] Benetos A, Gardner JP, Zureik M, Labat C, Xiaobin L, Adamopoulos C, Temmar M, Bean KE, Thomas F, Aviv A (2004). Short telomeres are associated with increased carotid atherosclerosis in hypertensive subjects. Hypertension.

[b15] Benetos A, Okuda K, Lajemi M, Kimura M, Thomas F, Skurnick J, Labat C, Bean K, Aviv A (2001). Telomere length as indicator of biological aging: the gender effect and relation with pulse pressure and pulse wave velocity. Hypertension.

[b16] Bischoff C, Petersen HC, Graakjaer J, Andersen-Ranberg K, Vaupel JW, Bohr VA, Kølvraa S, Christensen K (2006). No association between telomere length and survival among the elderly and oldest old. Epidemiology.

[b17] Brouilette SW, Moore JS, McMahon AD, Thompson JR, Ford I, Shepherd J, Packard CJ, Samani NJ, West of Scotland Coronary Prevention Study Group (2007). Telomere length, risk of coronary heart disease, and statin treatment in the West of Scotland Primary Prevention Study: a nested case-control study. Lancet.

[b18] Brouilette S, Singh RK, Thompson JR, Goodall AH, Samani NJ (2003). White cell telomere length and risk of premature myocardial infarction. Arterioscler. Throm. Vasc. Biol.

[b19] op den Buijs J, van den Bosch PPJ, Musters MW, van Riel NA (2004). Mathematical modeling confirms the length-dependency of telomere shortening. Mech. Ageing Dev.

[b20] Butler MG, Tilburt J, DeVries A, Muralidhar B, Aue G, Hedges L, Atkinson J, Schwartz H (1998). Comparison of chromosome telomere integrity in multiple tissues from subjects at different ages. Cancer Genet. Cytogenet.

[b21] Cawthon RM (2002). Telomere measurement by quantitative PCR. Nucleic Acids Res.

[b22] Cawthon RM, Smith KR, O’Brien E, Sivatchenko A, Kerber RA (2003). Association between telomere length in blood and mortality in people aged 60 years or older. Lancet.

[b23] Cherkas LF, Aviv A, Valdes AM, Hunkin JL, Gardner JP, Surdulescu GL, Kimura M, Spector TD (2006). The effects of social status on biological ageing as measured by white-blood-cell telomere length. Aging Cell.

[b24] Coale AJ, Kisker EE (1986). Mortality crossovers: reality or bad data?. Population Studies.

[b25] Corti MC, Guralink JM, Ferruci L, Izmirlian G, Leveille SG, Pahor M, Cohen HJ, Pieper C, Havlik HJ (1999). Evidence for black-white crossover in all-cause and coronary artery disease mortality in an older population. The North Carolina EPESE. Am. J. Public Health.

[b26] Demissie S, Levy D, Benjamin EJ, Cupples LA, Gardner JP, Herbert A, Kimura M, Larson MG, Meigs JB, Keaney JF, Aviv A (2006). Insulin resistance, oxidative stress, hypertension, and leukocyte telomere length in men from the Framingham Heart Study. Aging Cell.

[b27] Elo IT, Preston SH (1994). Estimating African American mortality from inaccurate data. Demography.

[b28] Finch C, Crimmins EM (2004). Inflammatory exposure and historical changes in human life-spans. Science.

[b29] Fitzpatrick AL, Kronmal RA, Gardner JP, Psaty BM, Jenny NS, Tracy RP, Walston J, Kimura M, Aviv A (2007). Leukocyte telomere length and cardiovascular disease in the Cardiovascular Health Study. Am. J. Epidemiol.

[b30] Ford AB, Haug MR, Jones PK, Roy AW, Folmar SJ (1990). Race-related differences among elderly urban residents: a cohort study, 1975–1984. J. Gerontol. Soc. Sci.

[b31] Gardner JP, Kimura M, Chai W, Durrani JF, Tchakmakjian L, Cao X, Lu X, Li G, Peppas AP, Skurnick J, Wright WE, Shay JW, Aviv A (2007). Telomere dynamics in Macaques and humans. J. Geron Ser. A Biol. Sci. Med. Sci.

[b32] Gardner JP, Li S, Srinivasan SR, Chen W, Kimura M, Lu X, Berenson GS, Aviv A (2005). Rise in insulin resistance is associated with escalated telomere attrition. Circulation.

[b33] Haddy TB, Rana SR, Castro O (1999). Benign ethnic neutropenia: what is a normal absolute neutrophil count?. J. Lab. Clin. Med.

[b34] Hansson GK, Libby P (2006). The immune response in atherosclerosis: a double-edged sword. Nat. Rev. Immunol.

[b35] Harris SE, Deary I, MacIntyre A, Lamb KJ, Radhakrishnan K, Starr JM, Whalley LJ, Shiels PG (2006). The association between telomere length, physical health, cognitive ageing, and mortality in non-demented older people. Neurosci. Lett.

[b36] van der Harst P, van der Steege G, de Boer RA, Voors AA, Hall AS, Mulder MJ, van Gilst WH, van Veldhuisen DJ, MERIT-HF Study Group (2007). Telomere length of circulating leukocytes is decreased in patients with chronic heart failure. J. Am. Coll. Cardiol.

[b37] Higgins M, Province M, Heiss G, Eckfeldt J, Ellison RC, Folsom AR, Rao DC, Sprafka JM, Williams R (1996). NHLBI Family Heart Study: objectives and design.. Am. J. Epidemiol.

[b38] Honig LS, Schupf N, Lee JH, Tang MX, Mayeux R (2006). Shorter telomeres are associated with mortality in those with *APOE*ɛ4 and dementia. Ann. Neurol.

[b39] Jeanclos E, Schork NJ, Kyvik KO, Kimura M, Skurnick JH, Aviv A (2000). Telomere length inversely correlates with pulse pressure and is highly familial. Hypertension.

[b40] Kimura M, Barbieri M, Gardner JP, Skurnick J, Cao X, van Riel N, Rizzo MR, Paoliso G, Aviv A (2007). Leukocytes of exceptionally old persons display ultra-short telomeres. Am. J. Physiol. Regul. Integr. Comp. Physiol.

[b41] Kimura M, Hjelmborg J, vB, Gardner JP, Bathum L, Brimacombe M, Lu X, Christiansen L, Vaupel LW, Aviv A (2008). Short leukocyte telomeres forecast mortality: a study in elderly Danish twins. Am. J. Epidemiol.

[b42] Loria CM, Liu K, Lewis CE, Hulley SB, Sidney S, Schreiner PJ, Williams OD, Bild DE, Detrano R (2007). Early adult risk factor levels and subsequent coronary artery calcification. The CARDIA Study. J. Am. Coll. Cardiol.

[b43] Markides KS, Machalek R (1984). Selective survival, aging and society. Arch. Gerontol. Geriatr.

[b44] Martens UM, Zijlmans JM, Poon SS, Dragowska W, Yui J, Chavez EA, Ward RK, Lansdorp PM (1998). Short telomeres on human chromosome 17p. Nat. Genet.

[b45] Martin-Ruiz CM, Gussekloo J, van Heemst D, von Zglinicki T, Westendorp RG (2005). Telomere length in white blood cells is not associated with morbidity or mortality in the oldest old: a population-based study. Aging Cell.

[b46] Matsubara Y, Murata M, Watanabe K, Saito I, Miyaki K, Omae K, Ishikawa M, Matsushita K, Iwanaga S, Ogawa S, Ikeda Y (2006). Coronary artery disease and a functional polymorphism of hTERT. Biochem. Biophys. Res. Commun.

[b47] Mayr FB, Spiel AO, Leitner JM, Firbas C, Kliegel T, Jilma B (2007). Ethnic differences in plasma levels of interleukin-8 (IL-8) and granulocyte colony stimulating factor (G-CSF). Transl. Res.

[b48] McClelland RL, Chung H, Detrano R, Post W, Kronmal RA (2006). Distribution of coronary artery calcium by race, gender, and age: Results from the multi-ethnic study of atherosclerosis (MESA). Circulation.

[b49] Nawrot TS, Staessen JA, Gardner JP, Aviv A (2004). Telomere length and possible link to X chromosome. Lancet.

[b50] Njajou OT, Cawthon RM, Damcott CM, Wu SH, Ott S, Garant MJ, Blackburn EH, Mitchell BD, Shuldiner AR, Hsueh WC (2007). Telomere length is paternally inherited and is associated with paternal lifespan. Proc. Natl Acad. Sci. USA.

[b51] Okuda K, Bardeguez A, Gardner JP, Rodriguez P, Ganesh V, Kimura M, Skurnick J, Awad G, Aviv A (2002). Telomere length in the newborn. Pediatr. Res.

[b52] Otten MW, Teutsch SM, Williamson DF, Marks JS (1990). The effects of known risk factors on the excess mortality of Black adults in the United States. JAMA.

[b53] Phillips D, Rezvani K, Bain BJ (2000). Exercise induced mobilisation of the marginated granulocyte pool in the investigation of ethnic neutropenia. J. Clin. Pathol.

[b54] Pickoff AS, Berenson GS, Schlant RC (1995). Bogalusa Heart Study 20th Anniversary Symposium. Am. J. Med. Sci.

[b55] Preston SH, Elo IT, Rosenwaike I, Hill M (1996). African-American mortality at older ages: results of matching study. Demography.

[b56] Saretzki G, Sitte N, Merkel U, Wurm RE, von Zglinicki T (1999). Telomere shortening triggers p53-dependent cell cycle arrest via accumulation of G-rich single stranded DNA fragments. Oncogene.

[b57] Sitte N, Saretzki G, von Zglinicki T (1998). Accelerated telomere shortening in fibroblasts after extended periods of confluency. Free Radic Biol. Med.

[b58] Slagboom PE, Droog S, Boomsma DI (1994). Genetic determination of telomere size in humans: a twin study of three age groups. Am. J. Hum Genet.

[b59] Takubo K, Izumiyama-Shimomura N, Honma N, Sawabe M, Arai T, Kato M, Oshimura M, Nakamura K (2002). Telomere lengths are characteristic in each human individual. Exp Gerentol.

[b60] Tang W, Detrano RC, Brezden OS, Georgiou D, French WJ, Wong ND, Doherty TM, Brundage BH (1995). Racial differences in coronary calcium prevalence among high-risk adults. Am. J. Cardiol.

[b61] Tchirkov A, Lansdorp PM (2003). Role of oxidative stress in telomere shortening in cultured fibroblasts from normal individuals and patients with ataxia-telangiectasia. Hum. Mol. Genet.

[b62] Valdes AM, Andrew T, Gardner JP, Kimura M, Oelsner E, Cherkas LF, Aviv A, Spector TD (2005). Obesity, cigarette smoking, and telomere length in women. Lancet.

[b63] Vasan RS, Demissie S, Kimura M, Cupples LA, Rifai N, White C, Wang TJ, Gardner JP, Cao X, Benjamin EJ, Levy D, Aviv A (2008). Association of leukocyte telomere length with circulating biomarkers of the renin-angiotensin-aldosterone system: the Framingham Heart Study. Circulation.

[b64] Vasa-Nicotera M, Brouilette S, Mangino M, Thompson JR, Braund P, Clemitson JR, Mason A, Bodycote CL, Raleigh SM, Louis E, Samani NJ (2005). Mapping of a major locus that determines telomere length in humans.. Am. J Hum Genet.

[b65] Weng N (2001). Interplay between telomere length and telomerase in human leukocyte differentiation and aging. J. Leukoc. Biol.

[b66] Whittle J, Kressin NR, Peterson ED, Orner MB, Glickman M, Mazzella M, Petersen LA (2006). Racial differences in prevalence of coronary obstruction among men with positive nuclear imaging studies. J. Am. Coll. Cardiol.

[b67] Wing S, Onton KG, Stallard E, Hames CG, Tryoler HA (1985). The black/white mortality crossover: Investigation in a community-based study. J. Gerontol.

[b68] Yan LL, Liu K, Daviglus ML, Colangelo LA, Kiefe CI, Sidney S, Matthews KA, Greenland P (2006). Education, 15-year risk factor progression, and coronary artery calcium in young adulthood and early middle age: the Coronary Artery Risk Development in Young Adults study. JAMA.

[b69] Yancy CW (2005). Heart failure in African Americans. Am. J Cardiol.

[b70] Youngren K, Jeanclos E, Aviv H, Kimura M, Stock J, Hanna M, Skurnick J, Bardeguez A, Aviv A (1998). Synchrony in telomere length of the human fetus. Hum. Genet.

[b71] von Zglinicki T, Serra V, Lorenz M, Saretzki G, Lenzen-Grossimlighaus R, Gessner R, Risch A, Steinhagen-Thiessen E (2000). Short telomeres in patients with vascular dementia: an indicator of low antioxidative capacity and a possible risk factor?. Lab. Invest.

