# Supplementary material for: Leukocyte telomeres are longer in African Americans than in whites: the National Heart, Lung, and Blood Institute Family Heart Study and the Bogalusa Heart Study
Source: Aging Cell. 2008 Aug;7(4):451–8. doi: 10.1111/j.1474-9726.2008.00397.x (PMC2810865; doi:10.1111/j.1474-9726.2008.00397.x)
Supplement: Supplementary file 1 [file acel0007-0451-SD1.pdf]

APPENDIX (SUPPLEMENT)

TRF Length Analysis by the overlay method (Vasan et al., in press): Samples were digested overnight with restriction enzymes digest set, *Hinf* I (5.2 U)/*Rsa* I (5.2 U) (Roche). DNA samples (3 µg each) and DNA ladders (1 kb DNA ladder plus 23.1kb fragment of λ DNA/Hind III fragments (Invitrogen, Carlsbad, CA)) were resolved on a 0.5% agarose gel (20 cm x 20 cm) at 50 V (GNA-200 Pharmacia Biotech). After 16 hr, the DNA was depurinated for 15 min in 0.25 N HCl, denatured 30 min in 0.5 mol/L NaOH/1.5 mol/L NaCl and neutralized for 30 min in 0.5 mol/L Tris, pH 8/1.5 mol/L NaCl. The DNA was transferred for 1 hr to a positively charged nylon membrane (Roche) using a vacuum blotter (Boeckel Scientific, Feasterville, PA). The membranes were spotted at 4 sites with diluted telomeric probe [digoxigenin 3'-end labeled 5'-$(CCTAAA)_3$] and then hybridized at 65 ^{o}C with the probe overnight in 5 x SSC, 0.1% Sarkosyl, 0.02% SDS and 1% blocking reagent (Roche). The membranes were washed 3 times at room temperature in 2 x SSC, 0.1% SDS each for 15 min and once in 2 x SSC for 15 min. The digoxigenin-labeled probe was detected by the digoxigenin luminescent detection procedure (Roche) and exposed on X-ray film. After scanning the TRF signal by densitometry, the membrane was stripped and re-probed with a molecular weight marker probe. The superimposition of the autoradiogram of the TRFs and the molecular weight ladder, using the 4 spotted sites of telomeric probe (arrows), yields the image used to calculate the mean TRF length (LTL), which was calculated as follows: TRF $=\sum OD_i/\sum(OD_i/MW_i)$, where OD_i is optical density at a given position in the lane and MW_i is molecular weight at that position. This formula accounts for the fact that longer

ScholarOne, 375 Greenbrier Drive, Charlottesville, VA, 22901 Support: (434) 817 2040 ext. 167
For Peer Review

telomeres bind more labeled probe and consequently appear darker on the X-ray film.

The position of each band of the MW ladder (y) was determined by $y= a_0+a_1*exp^{(-kb/a2)}$.

Appendix, Figure 1: Relationship between LTL, obtained using *Hph* I/*MnI* I vs. LTL obtained using *Hinf* I/*Rsa* I.

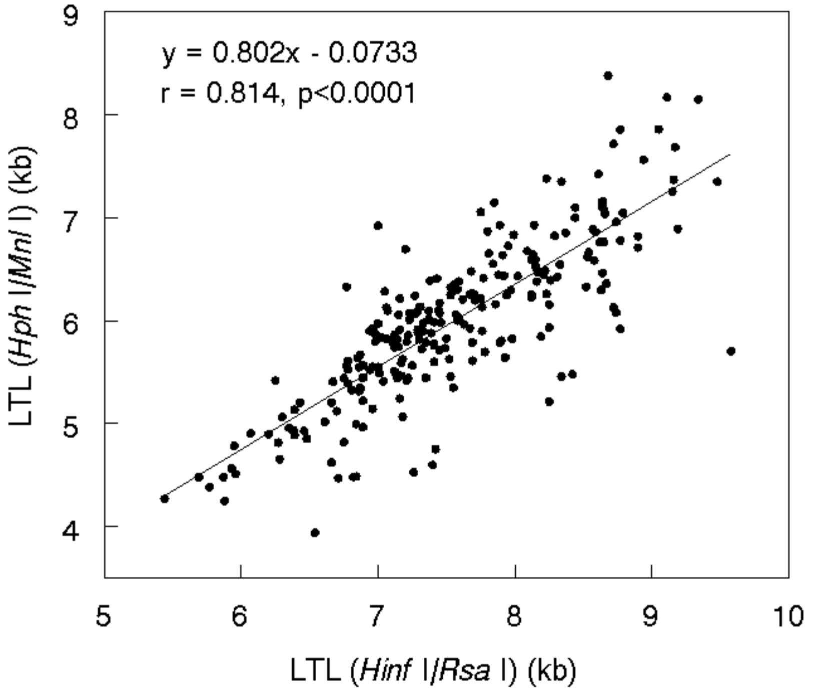

ScholarOne, 375 Greenbrier Drive, Charlottesville, VA, 22901 Support: (434) 817 2040 ext. 167

Appendix, Table 1: Leukocyte differential counts in a subset of the BHS by race and sex

	Whites			African Americans			Race Difference	
Cells/μL	Men	Women	p	Men	Women	p	Men	Women
	(n=120)	(n=178)		(n=37)	(n=81)			
Leukocytes	6473±172	6789±141	0.138	5241±195	6265±228	0.006	<0.001	0.057
Neuterophils	3883±146	4160±126	0.112	2802(142)	3488±187	0.030	<0.001	0.004
Lymphocytes	1810± 45	1960± 43	0.026	1716±105	2140±74	0.007	0.355	0.028
Basophils	22±2	20±1	0.357	26±5	29±4	0.818	0.385	0.016
Eosinophils	202±11	162±8	0.002	181±16	123±9	0.003	0.343	0.004
Monocytes	556±14	486±11	<0.001	516±25	485±19	0.346	0.184	0.946

For Peer Review

ScholarOne, 375 Greenbrier Drive, Charlottesville, VA, 22901 Support: (434) 817 2040 ext. 167
